# A Rapid Screening Analysis of Antioxidant Compounds in Native Australian Food Plants Using Multiplexed Detection with Active Flow Technology Columns

**DOI:** 10.3390/molecules21010118

**Published:** 2016-01-20

**Authors:** Emmanuel Janaka Rochana Rupesinghe, Andrew Jones, Ross Andrew Shalliker, Sercan Pravadali-Cekic

**Affiliations:** 1 Food Science and Technology Research Group, School of Science and Health, Western Sydney University (Hawkesbury), Hawkesbury 1797, Australia; j.rupesinghe@westernsydney.edu.au; 2 Australian Centre for Research on Separation Sciences (ACROSS), School of Science and Health, Western Sydney University (Parramatta), Room LZ.G.73, Corner of Pemberton Street and Victoria Road, Parramatta 2150, Australia; 18209352@student.uws.edu.au (A.J.); r.shalliker@westernsydney.edu.au (R.A.S.)

**Keywords:** multiplexed detection, high performance liquid chromatography (HPLC), active flow technology (AFT), post-column derivatisation (PCD), antioxidants, lemon myrtle, cinnamon myrtle, native Australian food plants

## Abstract

Conventional techniques for identifying antioxidant and phenolic compounds in native Australian food plants are laborious and time-consuming. Here, we present a multiplexed detection technique that reduces analysis time without compromising separation performance. This technique is achieved using Active Flow Technology-Parallel Segmented Flow (AFT-PSF) columns. Extracts from cinnamon myrtle *(Backhousia myrtifolia*) and lemon myrtle (*Backhousia citriodora*) leaves were analysed via multiplexed detection using an AFT-PSF column with underivatised UV-VIS, mass spectroscopy (MS), and the 2,2-diphenyl-1-picrylhydrazyl (DPPH^•^) derivatisation for antioxidants as detection methods. A number of antioxidant compounds were detected in the extracts of each leaf extract.

## 1. Introduction

Native Australian food plants have been gaining interest over the last decade in the search for new novel functional foods. Netzel *et al.* (2006) [1] demonstrated that the total reducing capacity of five native Australian fruits was 3.5–5.4 times greater than that of blueberries (*Vaccinum* spp. *cv. Biloxi*). In a further study, six of the twelve fruits analysed had a total phenolic content 2.5–3.9 times greater than blueberries, which can be correlated to higher antioxidant activity [2]. Similarly, high phenolic content and antioxidant capacity was observed in six native Australian herbs and spices. Tasmanian pepper leaf (*Tasmannia lanceolata*), lemon myrtle (*Backhousia citriodora*), and anise myrtle (*Syzygium anisatum*) had the highest antioxidant response, the source of which was concluded to have been cinnamic acids and flavonoids [3]. One issue with such studies is that the method of identifying antioxidant and phenolic compounds is labour-intensive and time consuming, involving complex sample preparation, analysis with colorimetric reagents (e.g., 2,2-diphenyl-1-picrylhydrazyl (DPPH^•^)) and subsequent sample fractionation using chromatography, often with mass spectrometric detection [1,2,3].

**Figure 1:**
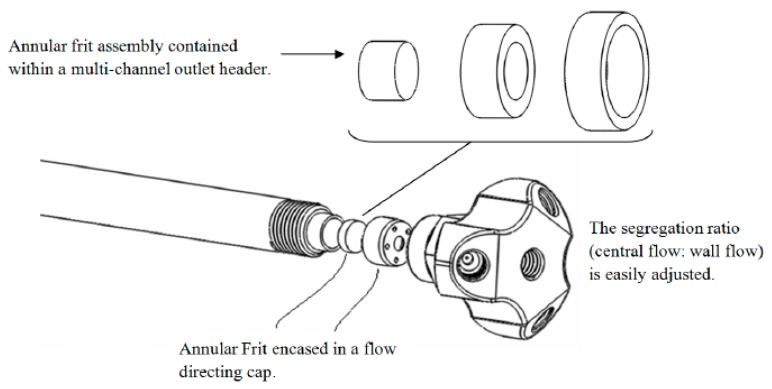
AFT-PSF column fitted with an annular 3 piece frit and multiport end fitting [10].

Active Flow Technology (AFT) High Performance Liquid Chromatography (HPLC) columns have been developed to overcome inefficiencies in separations associated with flow heterogeneity [4] and to increase separation efficiency and sensitivity, by establishing a wall-less “virtual” column within the actual column. This is achieved through a purpose-built four-port end-fitting and a three-component annular frit with an impermeable ring, which separates the wall and central flow regions of the column, without cross-flow, at the outlet of the column [4]. The design advantages of these columns have been comprehensively reviewed [4,5,6,7,8,9,10]. One column design within the AFT suite is termed the Parallel Segmented Flow (PSF) column, which provides the added advantage of enabling multiplexed detection protocols. The design of the AFT-PSF column is shown in Figure 1.

An important advantage of AFT-PSF columns is that each of the outlet exit ports can be connected directly to a detection source, thus enabling multiplexed detection without compromising separation performance, unlike traditional multi-detection setups such as split-flow or serial detection setup [11,12,13,14,15]. Pravadali-Cekic and Shalliker [15] have comprehensively reviewed the advantages of multiplexed detection using AFT-PSF columns compared to traditional methods of multi-detection techniques. The employment of AFT-PSF columns for multiplexed detection enables up to four detectors to be utilised simultaneously, all of which can be destructive, such as post-column derivatisation (PCD) reactions with subsequent detection or mass spectroscopy (MS). Additionally, with management of the flow ratios, flow rate limited detectors, such as MS, may be used with a higher column flow rate than if all of the mobile phase passed directly to the detector. Thus, multiplexed detection using PSF columns greatly reduces the analysis time, since one run yields multifaceted information and higher column flow rates can be utilised [11,12,13,14]. Furthermore, another advantage of the PSF column design is that it enables multiplexed detection to be undertaken with minimal additional post-column dead volume. Thus, smaller column formats and particles can be employed without compromising their efficiencies due to the extra column dead volume contributions. This factor further facilitates higher through-put opportunities.

In this study AFT chromatography columns in multiplexed detection mode were utilised to study the bioactivity of native Australian food plants (specifically, cinnamon myrtle *(Backhousia myrtifolia*), and lemon myrtle (*Backhousia citriodora*)) using a variety of detection techniques, namely UV-VIS absorbance, MS, and DPPH^•^ assays. The results stemming from these various detection techniques have been correlated and used to provide insight into the antioxidant properties of cinnamon myrtle and lemon myrtle leaf extracts, although this study is primarily concerned with the process of analysis and not so much the component character of the samples themselves.

## 2. Results and Discussion

### 2.1. Multiplexed Detection

The design of the end fitting of the AFT-PSF column allows for up to four effluent streams to be independently monitored with no detection delay. Additionally, multiple destructive techniques, such as MS and PCD reactions, can be employed at the same time increasing the amount of information that can be gathered from each injection. This results in time saving (compared to if each destructive technique needed to be performed using a separate injection) and also makes peak matching easier as the various detection techniques are not affected by random injection to injection errors. Finally, by manipulating the relative flow ratios between the central and peripheral streams at the outlet of the column, a high flow rate through the column can be used and then reduced for flow rate critical detectors such as MS. In this case the mobile phase flow rate through the column was 4.0 mL·min^−1^ producing back pressure close to the manufacturer’s suggested maximum pressure, and through the adjustment of flow ratio upon the column outlet, the flow to the MS detector was reduced to 1.0 mL·min^−1^ (close to the maximum flow rate that can be used in this detector when highly aqueous mobile phases are employed). Thus, the time saving here was around four-fold compared to the conventional technique where the flow rate through the column would be limited to around 1.0 mL·min^−1^ in order to achieve the necessary flow restrictions suited to the MS detector. This does not account for the advantage also offered by being able to multiplex the detection process with minimal dead volume, thus enabling the use of short- to high-throughput column technology, or by not having to undertake separate injections per detection mode. If these factors are also considered the time saving for a three-detector process could be as much as 7 to 8 times.

#### 2.1.1. Underivatised UV-VIS Detection

**Figure 2:**
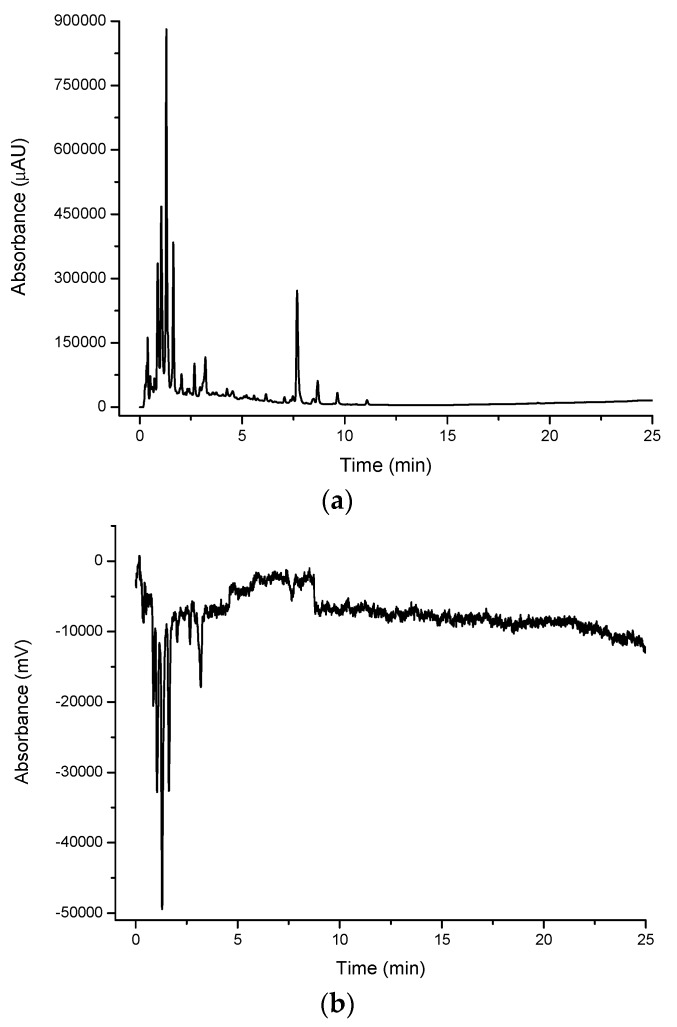

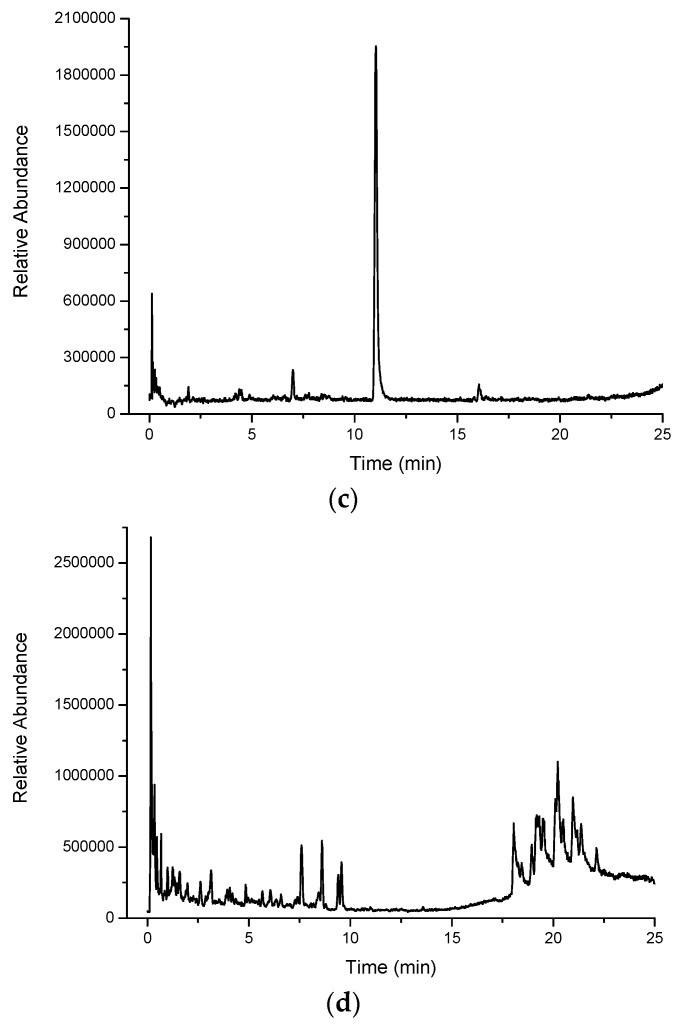
Multiplexed detection of cinnamon myrtle (CM) water extract with (**a**) UV set at 254 nm; (**b**) DPPH^•^; (**c**) MS-TIC positive scan mode; and (**d**) MS-TIC negative scan mode.

A large number of peaks were observed for all extracts when detected in their native (underivatised) form using UV-VIS detection. Figure 2a shows a large number of compounds eluting in the water extract for cinnamon myrtle. It is noted that the majority of the high intensity peaks that were observed in the cinnamon myrtle water extract eluted within the first 2 min of the chromatogram, indicating the presence of a large number of highly polar compounds. Furthermore, very few peaks were detected with retention times of greater than 10 min.

**Figure 3:**
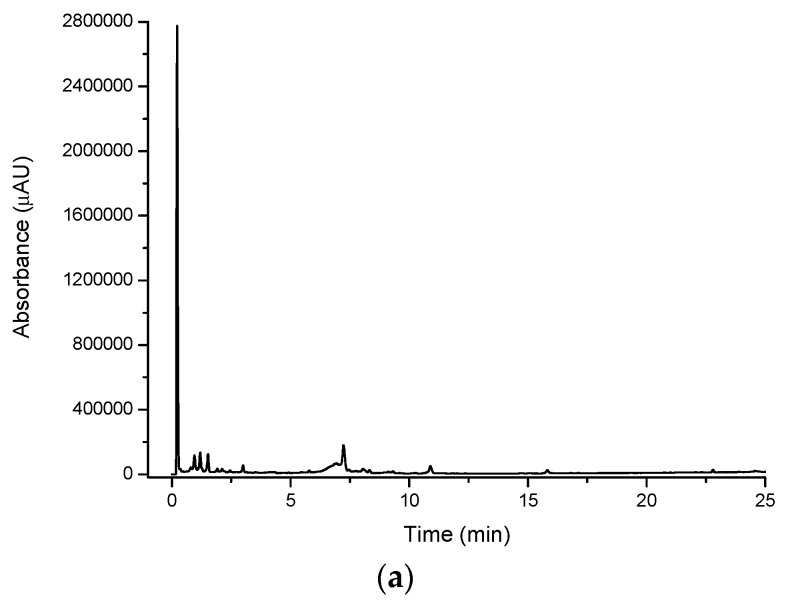

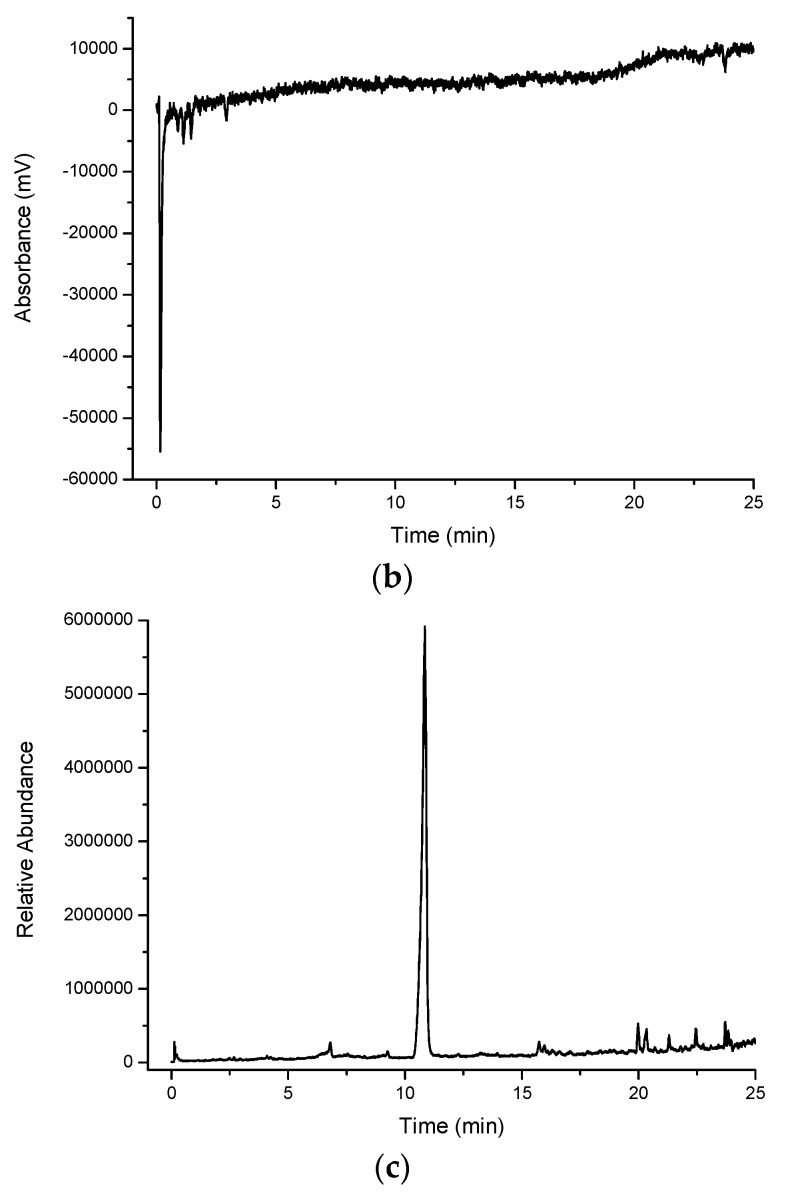
Multiplexed detection of cinnamon myrtle (CM) methanol extract with (**a**) UV set at 254 nm; (**b**) DPPH^•^; and (**c**) MS-TIC positive scan mode.

In contrast, the methanol extract of the cinnamon myrtle (Figure 3a) shows a single, very large peak eluting close to the void dominating the chromatogram. A number of secondary peaks can also be observed in the cinnamon myrtle methanol extract though they appear with lower intensity than the peaks observed in the water extract. Not surprisingly, the methanol extract for the cinnamon myrtle showed a greater abundance of compounds eluting with retention times of more than 5 min.

**Figure 4:**
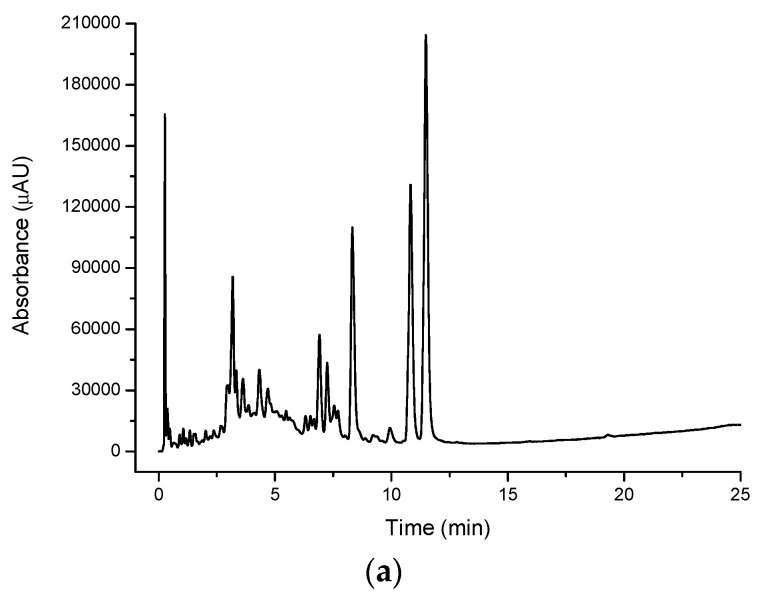

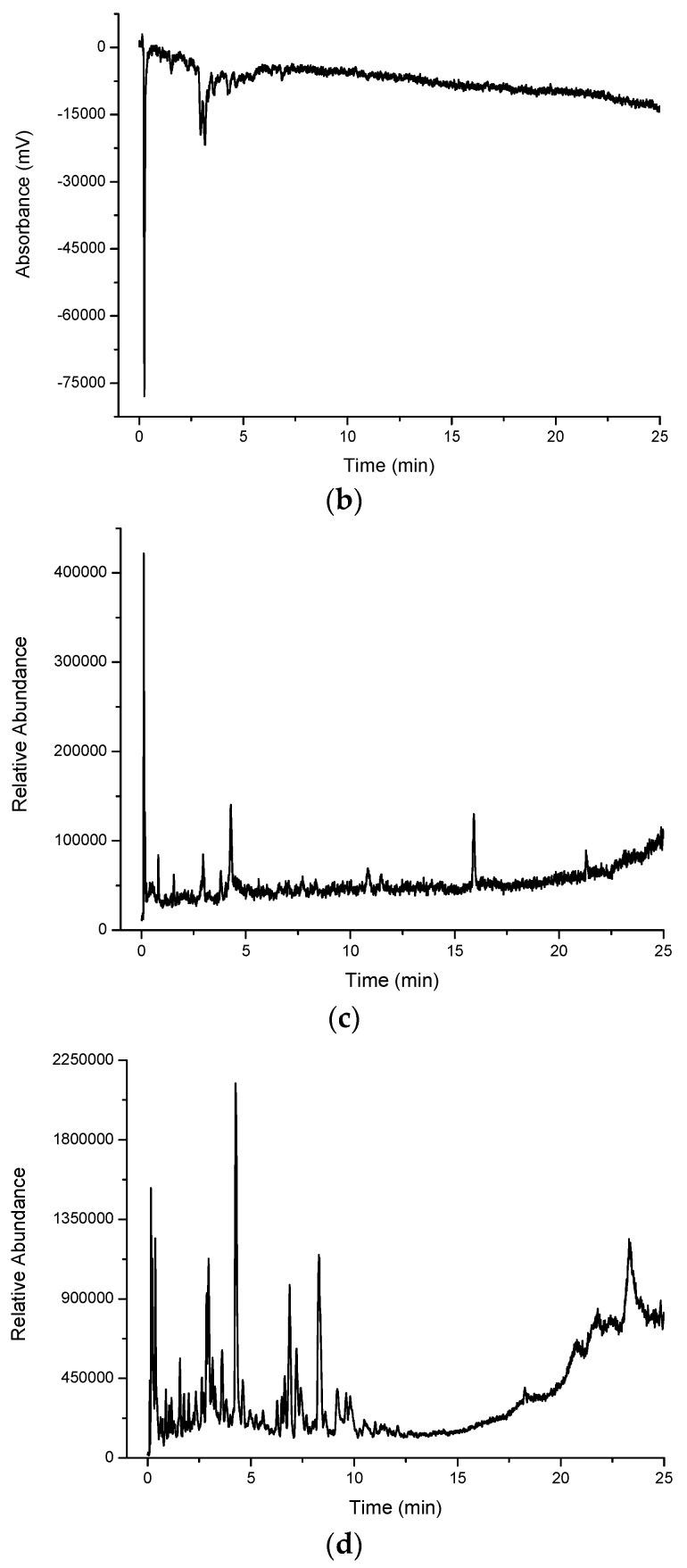
Multiplexed detection of lemon myrtle (LM) water extract with (**a**) UV set at 254 nm; (**b**) DPPH^•^; (**c**) MS-TIC positive scan mode; and (**d**) MS-TIC negative scan mode.

The chromatogram of the water extract from the lemon myrtle leaf (Figure 4a) shows a higher abundance of peaks eluting with a retention time of more than 5 min compared to the cinnamon myrtle water extract, with relatively few peaks with retention times of less than 3 min. Furthermore, the intensity of the peaks observed in the lemon myrtle extract was found to be significantly less than that in the cinnamon myrtle extract, since water is a weaker extraction solvent for the less polar compounds. Two of the highest intensity peaks in the lemon myrtle extract eluted close to each other with retention times of approximately 12 min.

**Figure 5:**
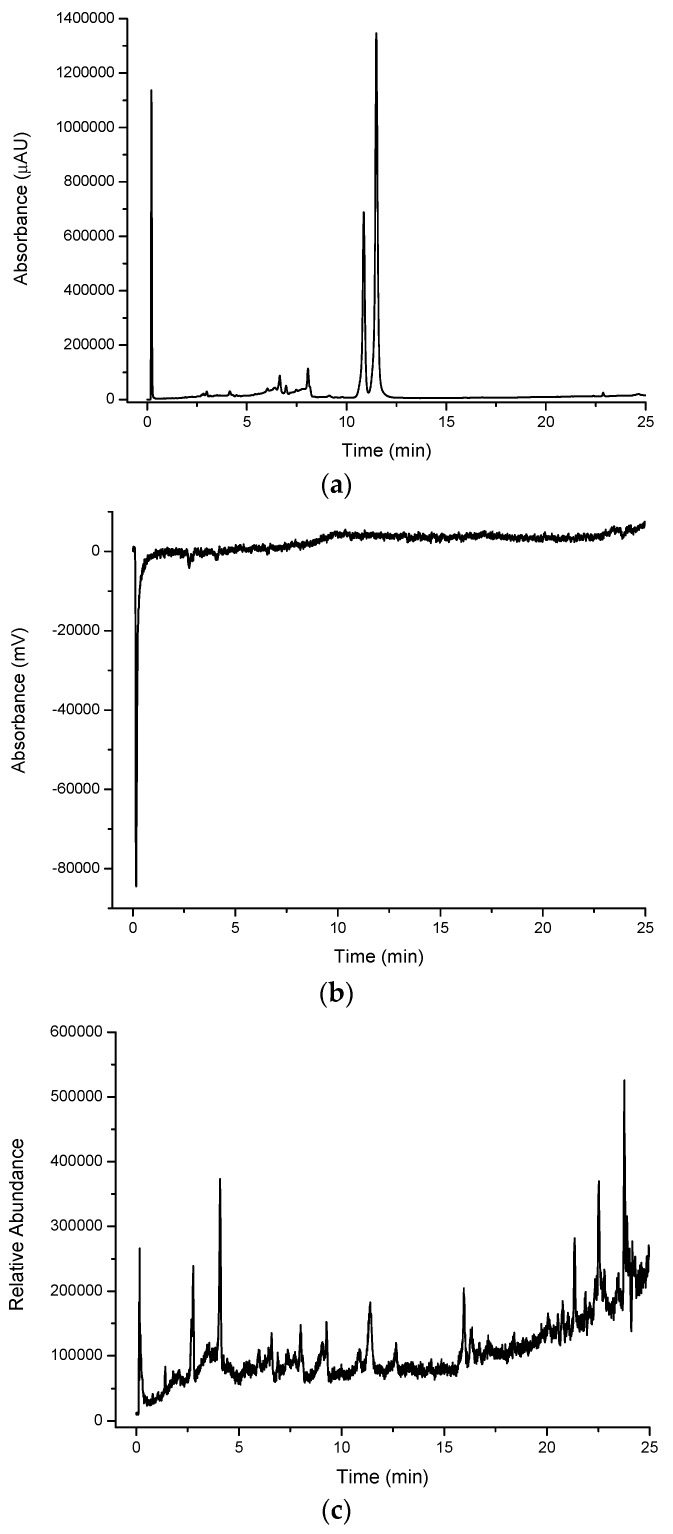
Multiplexed detection of lemon myrtle (LM) methanol extract with (**a**) UV set at 254 nm; (**b**) DPPH^•^; and (**c**) MS-TIC positive scan mode.

Figure 5a shows the underivatised UV-VIS response for the lemon myrtle methanol extract. Like the cinnamon myrtle, the lemon myrtle methanol extract shows less peaks compared to the water extract, however, a small number of these peaks elute with much greater intensity compared to the water extract, again not surprising since the less polar compounds will exhibit higher solubility in methanol compared to water. The chromatogram of the methanol extract of lemon myrtle is dominated by three intense peaks, namely a peak that eluted close to the void (likely to a mixture of numerous components) and the two large peaks that appear in the water extract that elute with retention times of approximately 12 min. Like in the water extract, there are few peaks that elute with retention times of less than 3 min, however, a cluster of peaks can be observed with retention times of between 6 and 8 min.

#### 2.1.2. DPPH^•^ Detection

The DPPH^•^ radical was used in the multiplexed setup as a selective detection technique for antioxidants Figure 2b, Figure 3b, Figure 4b and Figure 5b show the DPPH responses of each extract considered in the study. From these chromatograms it is clear that the majority of the antioxidant compounds appear within the first 5 min of the chromatogram indicating that the antioxidant compounds found in these extracts are polar compounds. Despite the lemon myrtle in particular showing a number of high intensity peaks in the underivatised UV-VIS chromatogram that elute later in the chromatogram, these peaks do not respond to DPPH^•^; therefore, it can be concluded that they are not antioxidants. Furthermore, it is also clear that the water extracts (Figure 2b and Figure 4b) show a greater abundance of compounds that respond to DPPH^•^ compared to the methanol extracts (Figure 3b and Figure 5b). Both of the methanol extracts, however, did show an increase in the intensity in the DPPH^•^ response of the peak eluting close to the void at 0.3 min.

Comparison of the water extract chromatograms of both leaves (Figure 2b and Figure 4b) shows a number of differences in the antioxidant profiles of both leaves. The lemon myrtle chromatogram shows the highest intensity single peak of either chromatogram. This peak, however, with a retention time of 0.3 min is very close to the void and therefore may be due to the presence of a large number of weakly-retained compounds. The cinnamon myrtle leaves, on the other hand, showed a larger number of retained antioxidants. Additionally, the retained antioxidant peaks had a higher intensity in the cinnamon myrtle extract compared to the lemon myrtle extract.

**Figure 6:**
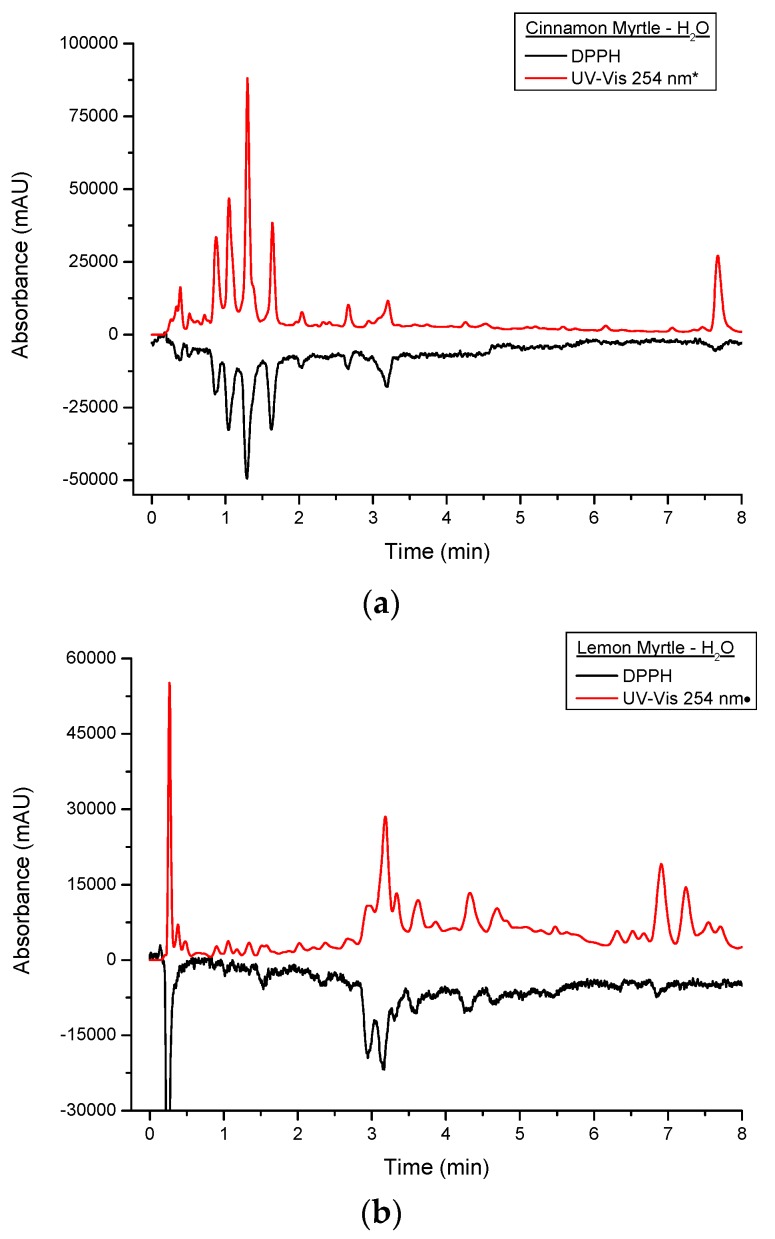
Close up of multiplexed detection DPPH^•^ and UV set at 254 nm of water extracts of (**a**) CM and (**b**) LM. UV-VIS response was appropriated by a *:10-fold decrease;•:three-fold decrease.

Figure 6 shows a comparison of the DPPH^•^ and underivatised UV-VIS responses from the first 8 min of the cinnamon myrtle and lemon myrtle water extract chromatograms. In Figure 6a, it can be seen that the majority of the peaks detected in the underivatised UV-VIS detector for the cinnamon myrtle extract also show a response to DPPH^•^. Only a handful of the smaller underivatised UV-VIS peaks were not detected after DPPH^•^ derivatisation, which could indicate that either, these smaller peaks were not due to antioxidants or that their concentration was less than the limit of detection for the DPPH^•^ derivatisation. Furthermore, the majority of the peaks detected in the cinnamon myrtle extract show similar relative response factors in both the underivatised UV-VIS and the DPPH^•^ chromatograms. The exception to this being the peak that eluted at 7.5 min, which does show a DPPH^•^ response, though it is much weaker compared to other peaks of comparable intensity in the underivatised UV-VIS chromatogram.

Figure 6b also shows that a number of peaks detected in the underivatised UV-VIS detector showed DPPH^•^ response in the lemon myrtle extract as well. However, compared to the cinnamon myrtle extract, the lemon myrtle extract shows greater variation between the underivatised UV-VIS and the DPPH^•^ responses. The trio of peaks that elute with retention times of around 3 min, for example, show very different relative responses in the underivatised UV-VIS and DPPH^•^ chromatograms. In the DPPH^•^ chromatogram the first two peaks show relatively similar response, while in the underivatised chromatogram the second peak is much higher intensity compared to the first. Furthermore, in the region between 6 and 8 min, a number of peaks appear in the underivatised UV-VIS chromatogram, however, in this region, there are only a couple of peaks that respond to DPPH^•^ and the response of these compounds is also relatively low compared with their underivatised UV-VIS responses.

**Figure 7:**
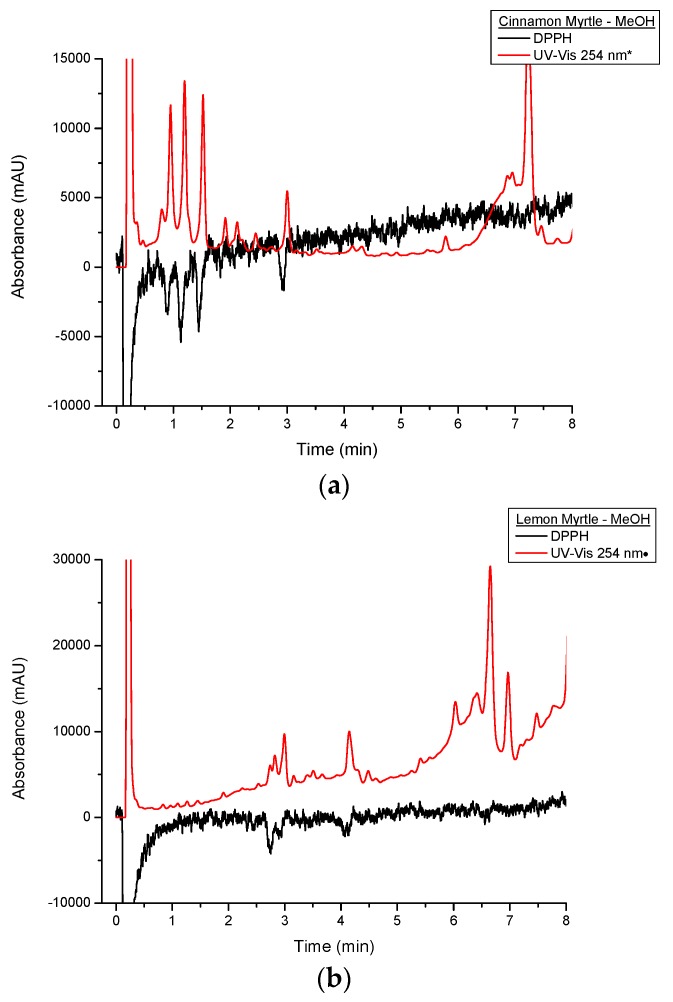
Close up of multiplexed detection DPPH^•^ and UV set at 254 nm of methanol extracts of (**a**) CM and (**b**) LM. UV-VIS response was appropriated by a *: 10-fold decrease;•: three-fold decrease.

Figure 7 shows a comparison of DPPH^•^ and underivatised UV-VIS responses of the first 8 min of the cinnamon myrtle and lemon myrtle methanol extract chromatograms. As has previously been discussed, the methanol extracts show lower DPPH^•^ response compared to the water extracts. Like the water extracts, peaks that show a response to DPPH^•^ can also be seen in the underivatised UV-VIS chromatograms. However, there are more high intensity peaks in the methanol extracts that do not respond to DPPH^•^ compared to the water extracts, particularly in the region after 5 min. These results would indicate that not only does water extract the antioxidants out of the leaves more effectively compared to methanol, it also does not extract a number of compounds that are not antioxidants making it the better choice of solvent for the analysis of antioxidants in these leaves.

#### 2.1.3. Mass Spectroscopy Detection

MS data was obtained using the positive scan mode for all of the extracts considered in the study. Compared to the underivatised UV-VIS and DPPH^•^ chromatograms, the positive scan mode MS chromatograms show very different profiles (Figure 2c, Figure 3c, Figure 4c and Figure 5c). The cinnamon myrtle chromatograms (Figure 2c and Figure 3c) are dominated by a peak that elutes with a retention time of 11 min. This peak does not appear in either the underivatised UV-VIS or DPPH^•^ chromatograms. A small number of secondary peaks occur in both chromatograms, although they too do not appear to correspond to any major peak in either the underivatised UV-VIS or DPPH^•^ chromatograms.

Unlike the cinnamon myrtle, the negative scan MS chromatograms of the lemon myrtle are not dominated by a single peak, but show a number of peaks with similar intensity. Additionally, some of these peaks match in retention time to peaks that appear in the underivatised UV-VIS and DPPH^•^ chromatograms. In particular, peaks with retention times of 3 and 4.5 min appear in a similar area of the chromatogram to peaks that respond to both underivatised UV-VIS and DPPH^•^. Additionally, the large peaks at around 12 min in the underivatised UV-VIS chromatograms appear as small peaks in the MS negative scan.

As the number of peaks identified in the MS in positive scan mode was smaller than expected, the multiplexing experiment was repeated for the water extracts using both negative and positive MS scan modes. The water extracts were chosen as they showed the greatest number and intensity of antioxidant peaks in the DPPH^•^ chromatograms. Both cinnamon myrtle and lemon myrtle showed a greater abundance of peaks in negative scan mode compared to positive scan mode. Additionally, both chromatograms showed a large number of peaks eluting within the first 5 min of the chromatogram where the majority of the compounds that gave a response to DPPH^•^ eluted.

Table 1 shows the peaks that were detected in the DPPH^•^ chromatograms along with the masses of those peaks as determined in the MS scans and possible identification based on the MS data. Due to the non-specificity of the MS scan, a number of the peaks that were identified in the MS data were due to more than one major *m*/*z* value indicating the presence of two co-eluting species being detected. Furthermore, a number of peaks that were detected in the DPPH^•^ chromatograms did not show peaks in the MS data, indicating that these species did not ionise in the MS conditions used in the method. Additionally, it can be seen that a number of peaks that eluted had a *m*/*z* ratio that is either lower than typically observed in antioxidants, such as the peak at 0.5 min in the cinnamon myrtle water extract, or higher than that of typical antioxidants, such as the peaks at 3.3 and 4.6 min in the lemon myrtle water extract [3,16,17]. This indicates that two different peaks may be eluting at these retention times, one of which is observed in the DPPH^•^ chromatogram and the other that is observed in the MS chromatogram. Finally, it can be seen that where MS peaks were evident in both positive and negative scan modes, most of the peaks observed had very different *m*/*z* ratios in each mode, indicating the presence of co-eluting species.

From the MS data it is possible to perform some investigation into the identification of the antioxidants that were present in the extracts. The *m*/*z* ratio of each of the peaks identified in the MS chromatograms was compared to the molecular masses of known antioxidants. If a match between the *m*/*z* ratio of the peak and the molecular mass of one or more antioxidants was found, this was considered a possible identification for that peak. For example, both lemon myrtle extracts show peaks with a *m*/*z* ratio of 139 Da indicating that the peak may be due to hydroxybenzoic acids. However, positive identification is impossible without additional information such as MS/MS data and/or the comparison of the peaks with standard solutions. Due to the non-specific nature of the MS data that was collected in the study, only preliminary identification of the peaks could be performed. Thus a number of peaks could either not be identified or were identified as one of a number of possible antioxidants.

**Table 1:** List of peaks observed in the DPPH^•^ chromatograms and the masses of those peaks as determined from the MS data and possible identification based on the MS data. Where two significant masses were observed in the MS data, the first mass listed is the most abundant mass.

DPPH Peak-Retention Time (min)	Positive Scan *m*/*z*	Negative Scan *m*/*z*	Possible Identification
**Lemon Myrtle H_2_O Extract**			
0.2	176.76	132.69	Gallocatechin, Epigallocatechin, Taxifolin
1.5	187.73	304.94	Cyanidin 3-rutinoside
2.3	No peak	593.26, 394.93	Cyanidin 3-rutinoside
2.7	No peak	593.22	Hydroxybenzoic acids, Epicatechin, Gallocatechin, Taxifolin
2.9	138.71, 306.83	304.87	
3.1	No peak	935.25, 467.17	
3.3	No peak	865.33	
3.6	No peak	577.27, 407.04	Procyanidin B1, Procyanidin B2
4.3	290.78, 138.80	288.89	Catechin, Epicatechin, hydroxybenzoic acids
4.6	No peak	865.31	
6.9	No peak	463.11	Hyperoside
**Cinnamon Myrtle H_2_O Extract**			
0.4	172.81	172.75	
0.5	79.91	No peak	
0.9	No peak	No peak	
1.0	No peak	No peak	
1.3	No peak	No peak	
1.6	No peak	203.87	
2.0	No peak	203.85	
2.7	No peak	164.77	*p*-Coumaric acid
3.2	No peak	124.83	
7.6	No peak	302.75	Delphinidin-hexose-pentose, Taxifolin, Quercetin
**Lemon Myrtle MeOH Extract**			
0.2	380.82		
2.7	290.78, 138.77		Catechin, Epicatechin, Hydroxybenzoic acids
2.9	138.76, 306.82		Hydroxybenzoic acids, Epicatechin, Gallocatechin
4.0	290.79		Catechin, Epicatechin
**Cinnamon Myrtle MeOH Extract**			
0.2	380.92		
0.9	No peak		
1.1	No peak		
1.5	No peak		
2.9	No peak		

## 3. Experimental Section

### 3.1. Chemicals and Reagents

HPLC grade solvents were supplied by (Themo Fisher Scientific, North Ryde, NSW, Australia). Milli-Q water was prepared in house using a 0.2 µm filter (Ultrapure, Millipore, Kilsyth, VIC, Australia). The DPPH^•^ (Sigma Aldrich-Castle Hill, NSW, Australia) reagent was prepared at a concentration of 0.1 mM in methanol with 0.1% formic acid, sonicated for 10 min and wrapped in foil to prevent exposure to light.

Mature cinnamon myrtle and lemon myrtle leaves were harvested from established trees grown in an uncontrolled environment at Muru Mittigar culture centre gardens in Castlereagh, NSW, Australia. The freshly harvested leaves were pounded separately in either Milli-Q water or methanol using a ceramic mortar and pestle. A total of 20 mL of solvent was added per gram of sample. The samples were sonicated for 5 min at room temperature and filtered using a 0.22 µm nylon filter.

### 3.2. Instrumentation and Chromatographic Conditions

#### 3.2.1. Column

Separations for multiplexed detection were conducted using a HyPURITY C18 50 × 4.6 mm, 3 µm column with a four-port outlet head-fitting (Thermo Scientific, Runcorn, Cheshire, UK).

#### 3.2.2. Multiplexed Detection Equipment

The chromatographic experiments were carried out using a Thermo UHPLC system coupled with a TSQ Vantage mass spectrometer equipped with HESI II source (Thermo Scientific, San Jose, CA, USA). The LC component was a Dionex Ultimate 3000 equipped with a quaternary pump, auto injector with an in-line degassing unit, and a RS diode array detector. The Thermo Vantage TSQ was operated as supplied from the manufacturer. An additional HPLC system (Shimadzu Kyoto, Kyoto, Japan) equipped with a Shimadzu LC-10AT*vp* pump, a Degassex model DG-440 inline degasser unit (Phenomenex, Torrance, CA, USA) and Shimadzu SPD-10Avp UV-VIS detector (Shimadzu, Rydalmere, NSW, Australia) was used for the DPPH detection system.

**Figure 8:**
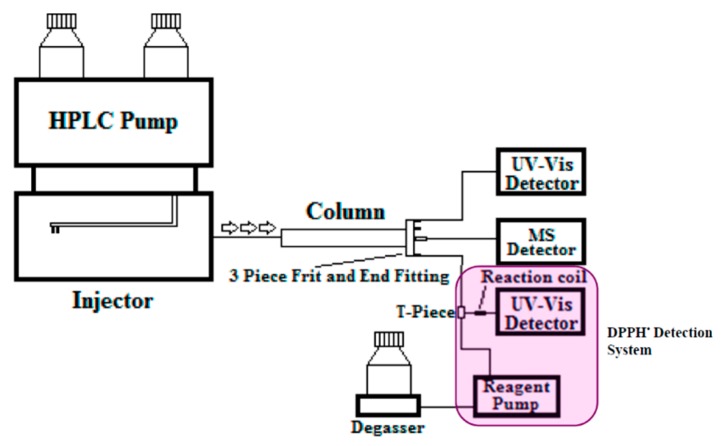
Multiplexed detection set up using an AFT-PSF column with central flow to MS, peripheral flow 1 to UV-VIS detector, and peripheral flow 2 combined to DPPH^•^ reagent flow for detection.

The flow ratios through the four exit ports of the AFT-PSF column was set to: 25% of the total flow rate from the central port, which was connected to the MS detector, 25% of the flow from peripheral port 1 (connected to the UV-VIS detector) and 50% of the total flow from peripheral port 2, which was connected to a DPPH^•^ detector via a zero dead volume T-piece and a 20 µL reaction coil. Peripheral port 3 was unused and blocked. Figure 8 illustrates the multiplexed detection set up with UV, MS, and DPPH^•^ detection.

The chromatographic conditions of the AFT-PSF column involved gradient elution with an initial mobile phase of 100% water (0.1% formic acid) running to a final mobile phase of 100% methanol (0.1% formic acid), at a rate of 4% min^−1^ then held for 1.5 min at 100% methanol. The flow-rate was set to 4 mL·min^−1^ and the injection volume was 20 µL. Figure 8 illustrates the multiplexed instrumental setup.

### 3.3. Detection Protocol

#### 3.3.1. Underivatised UV-VIS Detection

UV-VIS absorbance was obtained at a wavelength of 254 nm.

#### 3.3.2. Mass Spectrometry

MS was conducted using electrospray ionisation in positive and negative modes. A full scan detection method was used with Total Ion Count (TIC) analysis carried out on the TSQ Vantage mass spectrometer equipped with HESI II source under the following conditions: Vaporiser temperature 500 °C, capillary temperature 350 °C, sheath gas set at a rate of 60 unites, auxiliary gas flow 40 and sweep gas flow at 5 units, and spray voltage 3.5 kV.

#### 3.3.3. DPPH^•^ Detection

The DPPH^•^ detection process required an additional HPLC system (Shimadzu) coupled to the Thermo UHPLC system. The flow exiting peripheral port 2 was combined with the DPPH^•^ flow stream at T-piece, after which, the combined eluent stream then entered a reaction coil (20 µL) and finally the Shimadzu UV-VIS detector. The UV-VIS detector was set to a wavelength of 520 nm to detect the excess DPPH^•^ within the eluent. Since the flow rate to the DPPH^•^ reagent detector was reduced, the volumetric flow of the DPPH^•^ reagent was reduced accordingly. Specifically, the eluent stream from peripheral port 2 had a flow rate of 2 mL·min^−1^, so the DPPH^•^ reagent flow rate was set to 3 mL·min^−1^, in order to maintain the 1:1.5, eluent stream:DPPH^•^ reagent ratio.

### 3.4. Data Processing

Data analysis was undertaken using Origin (OriginLab, Northampton, MA, USA) and Microsoft Excel (Microsoft, Redmond, WA, USA). For the chromatographic profiles of DPPH^•^ detection, the blank chromatographic profile was subtracted from the sample chromatographic profile.

## 4. Conclusions

Screening for bioactivity in such native Australian food products using conventional HPLC methods is time consuming and laborious. Using HPLC with multiplexed detection, incorporating specific detection using PCD processes enables fast and efficient antioxidant screening. Multiplexed detection protocols, involving multiple destructive detection techniques were employed. Multiple detection modes were utilised on the same injection and the flow rate through the column could be run higher than the maximum allowable flow rate of flow rate of the MS resulting in a large time saving. Although previous studies have identified antioxidant compounds in lemon myrtle, this is the first time such experiments have been conducted on cinnamon myrtle and the first time either leaf has been analysed using DPPH^•^ assays. Both samples showed a large number of antioxidants, however, additional identification is required to definitively state whether the antioxidants detected are the same compounds. Using the multiplexed detection technique presented in this paper, detection methods such as derivatisation methods targeting additional functional groups and MS/MS detection could be used in order to positively identify the peaks detected. To validate the findings of this study, such identification of the antioxidant compounds observed in cinnamon myrtle and lemon myrtle will be necessary.
